# High-Contrast Gratings based Spoof Surface Plasmons

**DOI:** 10.1038/srep21199

**Published:** 2016-02-16

**Authors:** Zhuo Li, Liangliang Liu, Bingzheng Xu, Pingping Ning, Chen Chen, Jia Xu, Xinlei Chen, Changqing Gu, Quan Qing

**Affiliations:** 1 Key Laboratory of Radar Imaging and Microwave Photonics, Ministry of Education, College of Electronic and Information Engineering, Nanjing University of Aeronautics and Astronautics, Nanjing, 211106, China; 2 State Key Laboratory of Millimeter Waves, Southeast University, Nanjing, 210096, China; 3 Department of Physics, College of Liberal Arts and Sciences, Arizona State University, 871504, USA

## Abstract

In this work, we explore the existence of spoof surface plasmons (SSPs) supported by deep-subwavelength high-contrast gratings (HCGs) on a perfect electric conductor plane. The dispersion relation of the HCGs-based SSPs is derived analyt- ically by combining multimode network theory with rigorous mode matching method, which has nearly the same form with and can be degenerated into that of the SSPs arising from deep-subwavelength metallic gratings (MGs). Numerical simula- tions validate the analytical dispersion relation and an effective medium approximation is also presented to obtain the same analytical dispersion formula. This work sets up a unified theoretical framework for SSPs and opens up new vistas in surface plasmon optics.

Surface plasmons (SPs) are the area of intense interest at optical frequencies^1^,^2^,^3^. Their ability to capture photons from far field into short wavelength surface excitations on metal surfaces offers the potential for controlling light on a deep-subwavelength scale. At lower frequencies (terahertz (THz) or microwave), SPs cannot be excited since the metals exhibit the behavior of perfect electric conductor (PEC). Fortunately, it has been demonstrated that a highly conducting surface can be periodically textured to support transverse magnetic (TM) polarized bound surface waves that in many respects mimic, or spoof surface plasmons in optical regime^4^,^5^,^6^. Since then, SSPs with subwavelength transverse confinement have been found both in periodically perforated plane surfaces^7^,^8^,^9^ and in a variety of structured waveguide configurations^10^,^11^. Deep-subwavelength metallic gratings (MGs) and their ultrathin form are representative platforms to support SSPs^12^,^13^,^14^,^15^,^16^,^17^,^18^.

However, the characteristics of field confinement on the surface of metals and the intrinsic ohmic losses of most real metals will lead to a non-negligible propagation loss of the SSPs in long range transmissions, especially at upper end of microwave frequencies and THz frequencies. Moreover, as mentioned by Park *et al.*^19^, corrugated metal films are disadvantageous when considering implementation of active devices since it is difficult to change the optical properties of the metals by some form of external modulation^19^,^20^. Apart from the MGs, high-contrast gratings (HCGs) consisting of Bragg stack of high-index and low-index material blocks with subwavelength thicknesses have been widely studied in recent years^21^,^22^,^23^ because the optical properties of dielectric materials can usually be modulated by using electro-optical, magneto-optical, or thermo-optical effects^24^. Many interesting physical phenomena and important applications in optoelectronic devices were discovered based on the HCGs, for example, vertical-cavity surface-emitting lasers, high-*Q* optical resonators and ultralow loss hollow-core waveguides^25^,^26^. In addition, using the resonant coupling of SPs to radiation modes through the HCGs on the surface of a thin metal slab makes it possible to implement novel surface plasmon resonance based functional devices^18^,^19^,^20^ at optical frequencies. However, whether the HCGs can support the propagation of SSPs or not, to the best of our knowledge, has not been covered yet.

In this work, we extend the concept of SSPs by demonstrating that HCGs on a PEC plane shown in Fig. 1(a) can also support the propagation of SSPs both in two and three dimensions. To illustrate our findings, the dispersion relation of the HCGs-based SSPs is first derived analytically by combining multimode network theory^27^,^28^ with rigorous mode matching method, which coincides in the form with that of the MGs-based SSPs. Furthermore, the field intensity distributions and the corresponding electric-force lines of the HCGs-based SSPs show strong similarities with those of the MGs-based SSPs based on the simulation results of the commercial software COMSOL Multiphysics^10^. In addition, numerical simulations and effective medium approximations^29^,^30^,^31^ are both employed to validate the analytical dispersion formula. Finally, the existence of SSPs supported by three-dimensional (3D) domino-like^10^ HCGs on a PEC plane is validated by dispersion relation and field distributions through simulations.

## Results

### Dispersion relation of 2D HCGs-based SSPs

**Figure 1:**
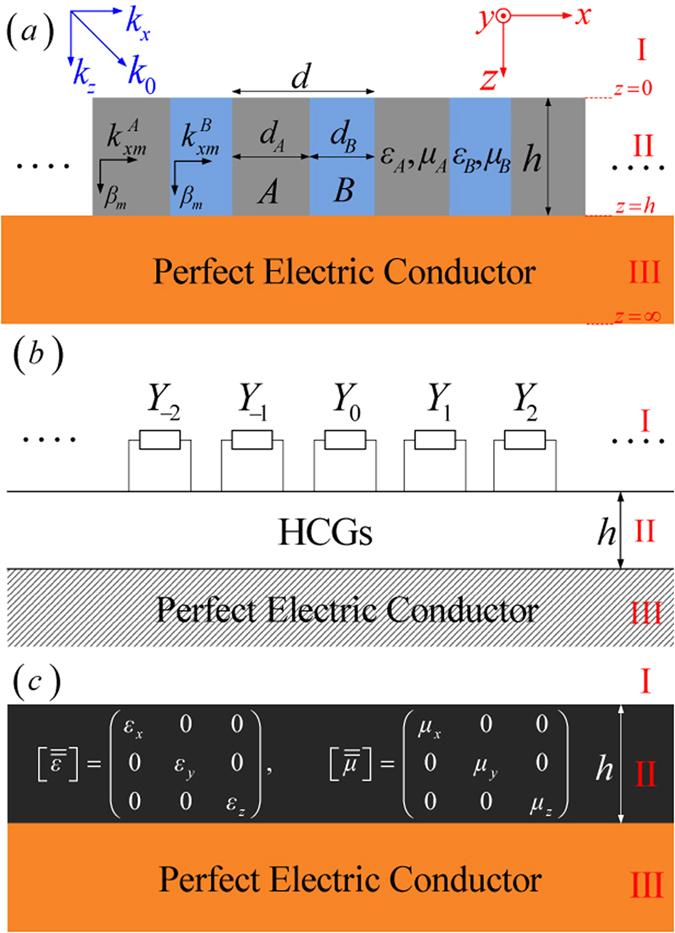
(**a**) The proposed 2D composite structure with one unit cell of height *h* and periodicity *d*. (**b**) Equivalent multimode network representation of the proposed structure in (**a**). (**c**) In the effective medium approximation the HCGs displayed in (**a**) behaves as a homogeneous but anisotropic layer of thickness *h* on top of a perfect electric conductor.

In Fig. 1(a), the proposed 2D composite structure is deep-subwavelength HCGs on a PEC plane with one unit cell composed of medium Blocks *A* and *B* with widths *d*_*A*_ and *d*_*B*_, relative permittivities 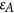 and 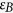 and permeabilities 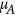 and 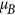. Since we are only interested in the surface electromagnetic (EM) modes supported by this structure, we first combine the multimode network theory with the rigorous mode matching procedure to derive the analytical dispersion formula of the HCGs-based SSPs based on a single-mode approximation.

We first assume that a TM-polarized plane wave with free space wavenumber 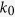 is incident upon the surface of the structure in Fig. 1(a) and Region I is air. According to the Floquet theorem, the relevant EM eigenmodes in Region II are a series of Bloch waves characterized by the longitudinal wave vectors 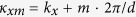 along the *x* direction and the perpendicular wave vectors 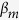 along the *z* direction with 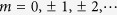. The field of each eigenmode also consists of an infinite set of space harmonics whose propagation factors along the *x* direction satisfy the Floquent condition 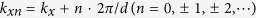. Meanwhile, due to the spatial periodicity of the HCGs in the *x* direction, the fields in Region I also contain an infinite set of space harmonics with the longitudinal wave vectors 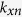 and the perpendicular wave vectors 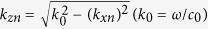. According to the multimode network theory, the eigenfunctions in Region I can be represented by an infinite number of transmission lines, each of which stands for one space harmonic. Thus, the whole structure can be modeled by an equivalent multimode network shown in Fig. 1(b).

To obtain the dispersion relation of this composite structure, we need to first solve a classical eigenmode problem when the HCGs region in the composite structure is regarded as an unbounded periodical array without the PEC and the air. Consequently, the fields in the unbounded HCGs can be expressed as a superposition of the obtained characteristic Floquet functions. Then, combining the multimode network theory with the Floquet condition, the dispersion relation of eigenmodes in the unbounded HCGs can be obtained^27^,^28^,^29^,^30^ as (see Supplementary Information S1)


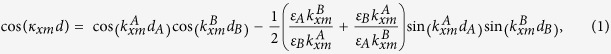


in which 
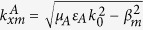
, 
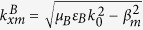
.

Since the eigenmodes in the unbounded HCGs have been determined, the transfer relationship of admittance matrix for each region can be obtained by applying the standard matching boundary conditions that the tangential components of the EM fields are continuous at the interface between adjacent regions. Then at the interface *z* = 0, the input admittance matrix **Y**_*dn*_ looking down into Region II can be obtained as


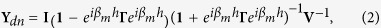


in which **Γ** = −**1** is the reflection coefficient matrix of the PEC plane and **1** stands for the unit matrix of infinite dimension. 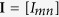 and 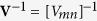 are the current amplitude matrix and the inverse of the voltage amplitude matrix for the *n*-th space harmonic through the Fourier expansion of the *m*-th mode current and voltage of the transmission lines respectively, which can be obtained by solving the eigenvalue problem of an unbounded HCGs.

Meanwhile, it is easy to determine the input admittance matrix 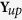 looking up into Region I at the interface *z* = 0, which is a diagonal matrix


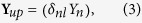


in which 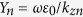 for the TM mode. Therefore, the complex eigenvalue of the composite structure can be finally determined using the generalized transverse resonance condition^27^,^28^ at *z* = 0 interface


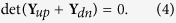


The determinant equation (4) defines the exact dispersion relation of the surface EM modes supported by the proposed structure. However, it involves matrices of infinite dimensions that must be truncated to get possible solutions. If we assume 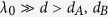, only the fundamental surface mode (*m* = 0) of equation (4) should be considered for obtaining the approximate dispersion relation. Thus, all the high-order diffraction effects can be safely neglected except the fundamental one (*n* = 0). For the case 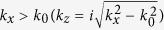, the dispersion relation of the HCGs-based SSPs can be derived as (see Supplementary Information S1)


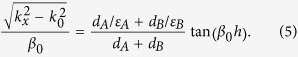


**Figure 2:**
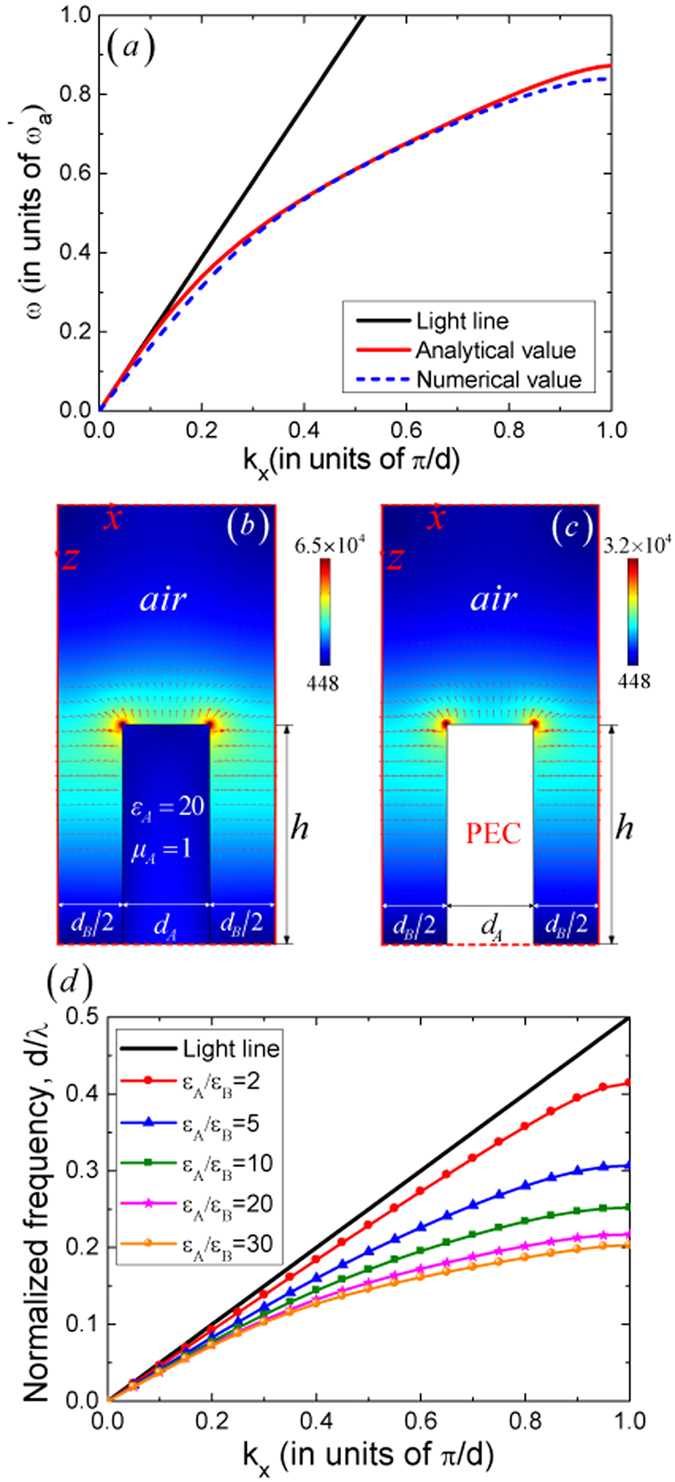
(**a**) The normalized dispersion relations for the fundamental HCGs-based SSP mode in a 2D composite structure, in which the medium and geometrical parameters of the HCGs are 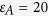, *μ*_*A*_ = 1, 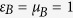, the height *h* = d and width 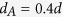, 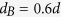, the metal is modeled as a PEC. (**b**,**c**) The distributions of the local electric field (V/m) and the corresponding electric-force lines (red arrows) on the *xz* plane when 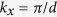 for the fundamental HCGs-based SSP mode and the fundamental MGs-based SSP mode respectively, in which the red solid and dotted lines stand for the Bloch and PEC boundaries respectively. (**d**) The normalized dispersion relations for the fundamental HCGs-based SSP mode in the 2D composite structure as a function of 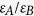.

By combining equations (1) and (5), we obtain and plot the dispersion curve as the red solid line in Fig. 2(a) for a particular case when 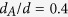, *h* = *d*, *ε*_*A*_ = 20, *μ*_*A*_ = 1, *ε*_*B*_ = *μ*_*B*_ = 1. For comparison, the simulated dispersion curve is obtained by the commercial software COMSOL Multiphysics, which is in excellent agreement with the analytical one and shown as the blue dotted line in Fig. 2(a). It is worth commenting on the coincidental form of the dispersion equations between HCGs-based SSPs and MGs-based SSPs. Equation (5) above and equation (14) in ref. 5 share the same form except that the denominator of the left-hand side term of the equation (5) is the perpendicular wave vector 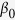 of the dominant mode in the terminal short-circuit cavity with dielectrics on both sides, which can be tuned by the geometrical and medium parameters of the HCGs. For the MGs-based SSPs, at large 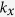, the asymptote frequency 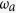 approaches 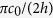-that is, the frequency location of a cavity waveguide mode inside the groove (in the limit 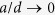, the locations of the different cavity waveguide modes correspond to the condition 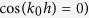, whereas for the HCGs-based SSPs in this work, the asymptote frequency 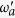 can be find by combining equation (1) with a similar condition 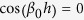 when 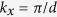, which also corresponds to the cavity waveguide mode inside the groove with dielectrics on both sides.

In order to gain a deeper insight into the behavior of the HCGs-based SSPs, we plot the distributions of the electric field 
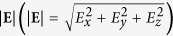
 and the corresponding electric-force lines (the red arrows) on the *xz* plane when 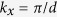 in Fig. 2(b,c), respectively. Nonzero electric field distributions can be found inside the dielectric for the HCGs-based SSPs, however, the field is relatively weak and in both cases, the electric fields are concentrated on the upper two corners of the dielectric and metallic gratings and decay exponentially away from the gratings. In addition, the distributions of the electric-force lines in the air region are nearly the same in both cases. All these characteristics demonstrate that the HCGs-based SSPs behave in the same way as the MGs-based SSPs do. Different from the MGs-based SSPs, this HCGs-based SSPs can be engineered by tuning both the geometrical and medium parameters. The normalized dispersion relations of the fundamental HCGs-based SSP mode in the 2D composite structure as a function of the contrast 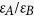 are shown in Fig. 2(d), which adds a degree of freedom to define and control the characteristics of this highly confined surface mode.

### Effective medium approximation

It is interesting to note that the same dispersion relation could also be obtained with effective medium approximations if we replace the HCGs with a single homogeneous but anisotropic medium layer of thickness *h* on top of the PEC surface (see the schematic drawing in Fig. 1(c)). The electric permittivity and magnetic permeability tensors of the effective homogeneous medium layer^29^ would have the following diagonal forms:


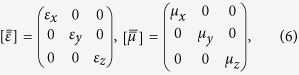


in which


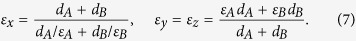


The only considered fundamental mode inside the HCGs can be characterized by the perpendicular wave vector 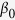. Thus, mapping the HCGs to an effective homogeneous medium means that the refraction index of the effective medium must be 
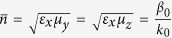
, leading to 
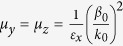
, 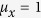. After some straightforward algebra, the specular reflection coefficient *R* for a TM-polarized plane wave impinging on the surface of a homogeneous medium layer of thickness *h* with the above effective EM parameters 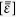 and 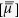 can be written as


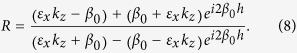


In the case when 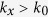, we can calculate the dispersion relation of the surface modes by looking at the zeroes of the denomination of *R* as (see Supplementary Information S2)


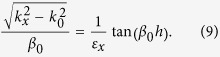


It is worth noting that this expression coincides with equation (5) in the limit when 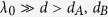 with the effective parameter 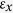 given in equation (7). Moreover, if we consider the limit 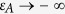 when Block *A* becomes a PEC, both the electric and magnetic fields inside Block *A* are exactly zero and the fundamental mode in the *AB* structure is just a transverse EM waveguide mode trapped only inside Block *B* with perpendicular wave vector 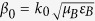. Thus, the dispersion relation of the HCGs-based SSPs in equation (5) will degenerate into the form of the MGs-based SSPs in ref. 5.

### HCGs-based SSPs in 3D structures

**Figure 3:**
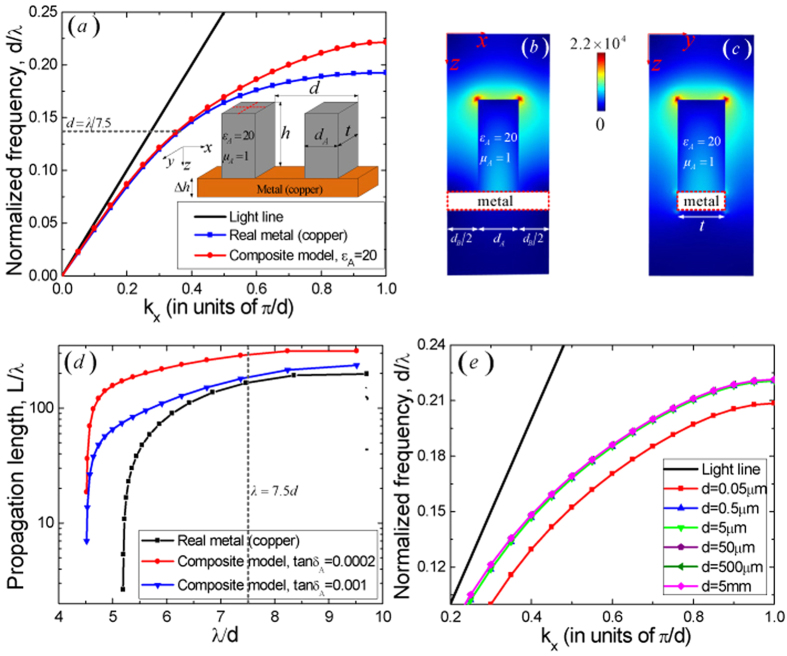
(**a**) The normalized dispersion relations of a 3D structure, in which the red solid line stands for the fundamental HCGs-based SSP mode and the blue solid line stands for the fundamental DSP mode. (Inset) Medium and geometrical parameters of the 3D structure, with 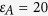, 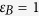 and 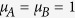, height *h* = *d*, thickness *t* = *d*, width 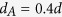, 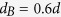, and the height of the metal copper plate 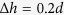. (**b**,**c**) The electric field distributions of the HCGs-based SSPs (V/m) on the *xz* and *yz* planes (the red dotted lines in (**a**)) when 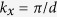 respectively. (**d**) The normalized propagation lengths of the fundamental HCGs-based SSP mode when tan*δ*_*A*_ = 0.0002 (the red solid line), tan*δ*_*A*_ = 0.001 (the blue solid line) and the fundamental DSP mode (the dark solid line), respectively. (**e**) Normalized dispersion relations with different values of *d* using a constant dielectric permittivity of the HCGs and copper optical constants with the same geometrical parameters in (**a**).

Finally, we address the issue whether SSPs also arise in 3D composite structure. Here we demonstrate that domino-like HCGs on a metal plate can also support the propagation of HCGs-based SSPs. A sketch of the proposed model is shown in the inset of Fig. 3(a), in which the geometrical parameters of the 3D HCGs are characterized by *h* = *d*, *d*_*A*_ = 0.4*d*, *d*_*B*_ = 0.6*d* and thickness *t* = *d* in the *y* direction and medium parameters of 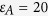, 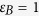, 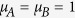. The metal plate also has the thickness *t* in the *y* direction and height 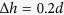 along the *z* axis. The normalized dispersion relation for the fundamental HCGs-based SSP mode is shown as the red line in Fig. 3(a) and the corresponding electric field distributions in the *xz* and *yz* planes are simulated and depicted in Fig. 3(b,c) respectively, which behave in the same way as domino surface plasmons (DSPs)^10^ (the dispersion relation of the fundamental DSPs is also given as the blue line in Fig. 3(a)) and demonstrate that a highly confined fields in both cross sections decay exponentially from the surface of the HCGs. In addition, the propagation lengths (*L* = *v*_*g*_/(2Im(*ω*)) (*v*_*g*_ is the group velocity, *ω* is the corresponding complex eigenfrequency and Im(*ω*) denotes the imaginary part of *ω*) of the fundamental HCGs-based SSP and normal DSP modes are calculated and shown in Fig. 3(d), in which the metal is represented by its actual dielectric function (copper in this case), as tabulated previously^32^ and the loss angle tangents of the HCGs are selected typically in the microwave frequencies as tan*δ*_*A*_ = 0.001 and tan*δ*_*A*_ = 0.0002 respectively. As expected, owing to the low loss of the HCGs, the HCGs-based SSPs has a larger propagation length (287*λ* and 180*λ* for tan*δ*_*A*_ = 0.0002 and tan*δ*_*A*_ = 0.001 respectively when *λ* = 7.5*d* with *d* = 5 mm) compared with the normal DSPs (165*λ* for *λ* = 7.5*d* with *d* = 5 mm), see the intersection points of three curves with the vertical gray dotted line in Fig. 3(d).

An important question concerning the frequency regimes the HCGs-based SSPs can operate in is depicted in Fig. 3(e), which shows the dispersion relations of the propagating HCGs-based SSP modes for the same geometric parameters as the inset in Fig. 3(a) with *t* = *d* but different values of *d*, ranging from 5 mm (*λ* = 6*d* = 30 mm, to operate in the microwave regime) to 0.05 *μ*m (*λ* = 6*d* = 0.3 *μ*m, to operate in the ultraviolet regime). To calculate these bands, the actual dielectric function of copper is also employed in the simulations. If the real part of the dielectric permittivity keeps unchanged, the normalized dispersion relations are not sensitive to the frequency regime of operation, which shows that HCGs-based SSPs exist at microwave, terahertz, mid-infrared and even ultraviolet frequencies. Although the permittivity of one dielectric could not keep constant in the whole spectrum, it is anticipated that we could find a certain dielectric in a certain frequency range with a similar permittivity thanks to the progress of advanced materials.

## Discussion

In conclusion, we have demonstrated the existence of SSPs supported by deep-subwavelength HCGs on a PEC plane both in 2D and 3D cases. Excellent agreement between the analytical and numerical dispersions of the HCGs-based SSPs validate our theory. The dispersion relation has further been verified through an effective medium approximation. The HCGs-based SSPs supported by this system have close resemblances with the MGs-based SSPs both in dispersion relation and field distributions. Thanks to this new concept, a whole bunch of phenomena already known to work for MGs-based SSPs can be safely transferred to HCGs-based SSPs. This finding may have a significant impact on both fundamental and applied research in surface plasmon optics.

## Methods

### Analytical derivations

The analytical dispersion relation of the HCGs-based SSPs in equation (5) is obtained by combining the multimode network theory with the rigorous mode matching procedure, in which the single-mode approximation is employed with only the fundamental surface mode (*m* = 0) considered and all high-order diffraction effects ignored. Equation (9) is derived through an effective medium approximation, in which the HCGs are replaced with a single homogeneous but anisotropic medium layer of thickness *h* and effective electric permittivity 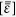 and magnetic permeability 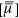 on top of the PEC surface. The details are given in the Supplementary Information.

### Numerical simulations

All the numerical dispersion curves, electric fields and the corresponding electric-force lines distributions are performed with the help of the Finite Element Method (FEM) using the commercial software COMSOL Multiphysics, in which the corresponding eigenvalue problem is posed in a single unit cell with Bloch boundary conditions used. For the simulation of ideal metals, PEC boundary conditions are employed. For realistic metals, the dielectric function of copper is used^10^,^32^. For the low loss HCGs in the microwave frequencies, the dielectric ceramics, such as MgTiO_3_ and CaTiO_3_^33^,^34^ with high dielectric permittivity and low loss angle tangent of less than 0.0001 can be introduced. At higher frequencies, from terahertz to ultraviolet frequencies, the dispersion relations in Fig. 3(e) could be implemented with a constant real part of dielectric permittivity of the HCGs.

## Additional Information

**How to cite this article**: Li, Z. *et al.* High-Contrast Gratings based Spoof Surface Plasmons. *Sci. Rep.* **6**, 21199; doi: 10.1038/srep21199 (2016).

## Supplementary Material

Supplementary Information
