# Supplementary material for: High-Contrast Gratings based Spoof Surface Plasmons
Source: Sci Rep. 2016 Feb 16;6:21199. doi: 10.1038/srep21199 (PMC4754736; doi:10.1038/srep21199)
Supplement: Supplementary Information [file srep21199-s1.pdf]

Supplementary Information

High-Contrast Gratings based Spoof Surface Plasmons

Zhuo Li[1,2,3,*,+], Liangliang Liu[1,+], Bingzheng Xu[1], Pingping Ning[1], Chen Chen[1], Jia Xu[1], Xinlei Chen[1], Changqing Gu[1] & Quan Qing[3]

[1] Key Laboratory of Radar Imaging and Microwave Photonics, Ministry of Education, College of Electronic and Information Engineering, Nanjing University of Aeronautics and Astronautics, Nanjing, 210016, China

[2] State Key Laboratory of Millimeter Waves, Southeast University, Nanjing, 210096, China

[3] Department of Physics, College of Liberal Arts and Sciences, Arizona State University, 871504, USA.

*Corresponding author: lizhuo@nuaa.edu.cn

[+] These two authors contributed equally to this work

1. Derivation of the dispersion relation of 2D HCGs-based SSPs with multimode network theory

Three steps should be taken to obtain the analytical dispersion relation of the HCGs-based SSPs. Firstly, the eigenfunctions are determined for Regions I (air) and II (HCGs). Secondly, boundary conditions are imposed at each interface to obtain the transfer relation of the admittance matrix between each region. Finally, the generalized transverse resonance technique is used.

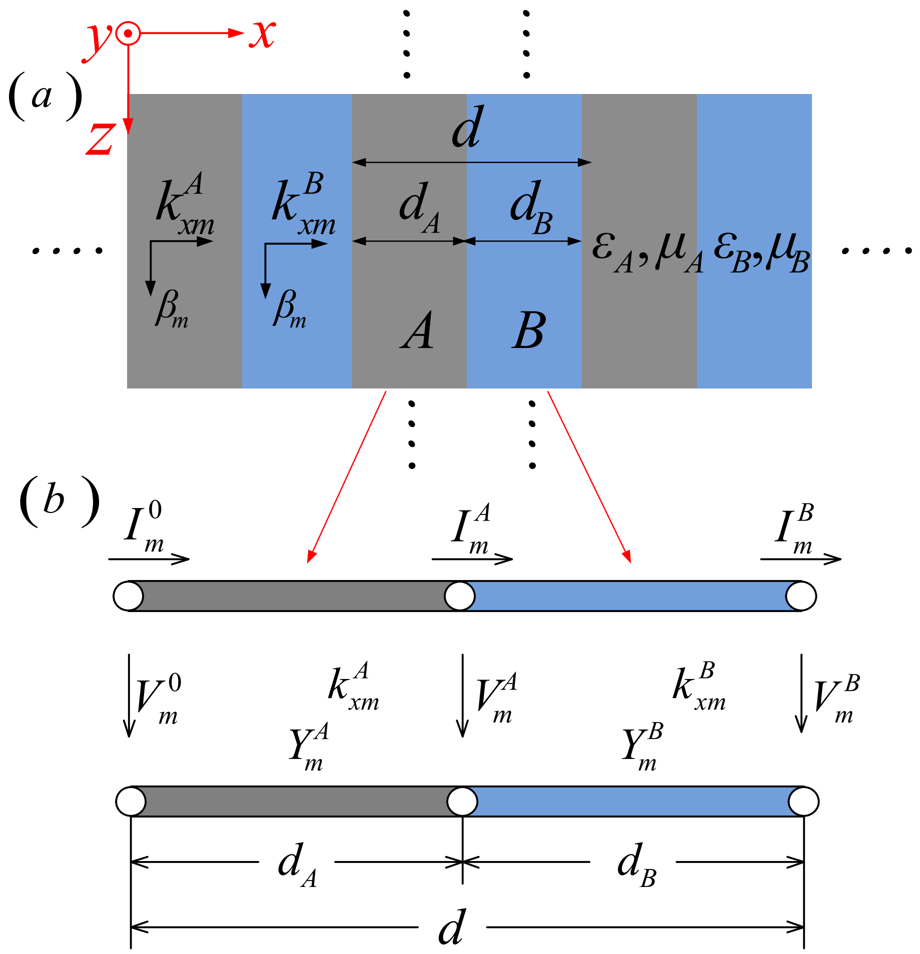

Supplementary Figure 1. (a) The unbounded periodic HCGs. (b) The equivalent transmission line of one unit cell in (a).

To determine the eigenfunctions of the HCGs region in Fig. 1(a) in the main text, the structure shown in **Supplementary Figure S1(a)** should be first analyzed, which is an unbounded periodic array. Two dielectric blocks in each unit cell are of widths d_A and d_B, relative permittivities ε_A, ε_B and permeabilities μ_A, μ_B. The equivalent circuit network for the m-th (m=0, ± 1, ± 2, $\cdots$) surface mode in one unit cell is shown in **Supplementary Figure S1(b)**. Thus, the eigenfunctions for the m-th TM mode can be expressed by the mode voltage and mode current as

$$H_{ym}^{i}\left(x,z\right)=I_{m}^{i}\left(x\right)e^{-j\beta_m z}, \tag{S1.1}$$

$$E_{xm}^{i}\left(x,z\right)=\frac{\beta_m}{\omega\varepsilon_0\varepsilon_i}I_{m}^{i}\left(x\right)e^{-j\beta_m z}, \tag{S1.2}$$

$$E_{zm}^{i}\left(x,z\right)=-V_{m}^{i}\left(x\right)e^{-j\beta_m z}, \tag{S1.3}$$

in which ε_0 is the permittivity of air, ε_i is the relative permittivity of Block i ($i=A,B$), ω is the angular frequency. Substituting equations (S1.1)-(S1.3) into Maxwell's Equations, we can find that the m-th surface mode voltage $V_{m}^{i}\left(x\right)$ and current $I_{m}^{i}\left(x\right)$ in Block i satisfy the following transmission line equations

$$\frac{dV_{m}^{i}\left(x\right)}{dx}=-jk_{xm}^{i}Z_{m}^{i}I_{m}^{i}\left(x\right), \tag{S1.4}$$

$$\frac{dI_{m}^{i}\left(x\right)}{dx}=-jk_{xm}^{i}Y_{m}^{i}V_{m}^{i}\left(x\right), \tag{S1.5}$$

where

$$Z_{m}^{i}=\frac{1}{Y_{m}^{i}}=\frac{k_{xm}^{i}}{\omega\varepsilon_0\varepsilon_i}, \tag{S1.6}$$

$$\left(k_{xm}^{i}\right)^2=\mu_i\varepsilon_i k_0^2-\beta_m^2. \tag{S1.7}$$

According to the Floquet theorem, we have

$$\begin{bmatrix} V_m^B \\ I_m^B \end{bmatrix} = \lambda \begin{bmatrix} V_m^0 \\ I_m^0 \end{bmatrix}. \tag{S1.8}$$

The input-output relation for the voltage and current column vector in one unit cell can be denoted by

$$\begin{bmatrix} V_m^B \\ I_m^B \end{bmatrix} = \mathbf{T}_m^B \begin{bmatrix} V_m^A \\ I_m^A \end{bmatrix}, \qquad \begin{bmatrix} V_m^A \\ I_m^A \end{bmatrix} = \mathbf{T}_m^A \begin{bmatrix} V_m^0 \\ I_m^0 \end{bmatrix}, \tag{S1.9}$$

in which the transmission matrix $\mathbf{T}_m^i$ for Block i can be expressed as

$$\mathbf{T}_m^i = \begin{bmatrix} \cos\left(k_{xm}^i d_i\right) & -jZ_m^i \sin\left(k_{xm}^i d_i\right) \\ -jY_m^i \sin\left(k_{xm}^i d_i\right) & \cos\left(k_{xm}^i d_i\right) \end{bmatrix}. \tag{S1.10}$$

Then, the total transformation relation in one unit cell can be denoted as

$$\begin{bmatrix} V_m^B \\ I_m^B \end{bmatrix} = \mathbf{T}_m^B \begin{bmatrix} V_m^A \\ I_m^A \end{bmatrix} = \mathbf{T}_m^B \mathbf{T}_m^A \begin{bmatrix} V_m^0 \\ I_m^0 \end{bmatrix} = \mathbf{T} \begin{bmatrix} V_m^0 \\ I_m^0 \end{bmatrix}, \tag{S1.11}$$

and the total transmission matrix $\mathbf{T}$ for one unit cell is of the form

$$\mathbf{T} = \begin{bmatrix} t_m^{AA} & t_m^{AB} \\ t_m^{BA} & t_m^{BB} \end{bmatrix}. \tag{S1.12}$$

Combining equations. (S1.8) and (S1.11), we can obtain the following equation

$$\left(\mathbf{T} - \lambda \mathbf{1}\right) \begin{bmatrix} V_m^0 \\ I_m^0 \end{bmatrix} = 0, \tag{S1.13}$$

in which $\mathbf{1}$ is the unit matrix.

According to the linear algebra theory, the existence of a nontrivial solution for equation (S1.13) requires that the determinant of the coefficient matrix vanishes, namely

$$\det\left(\mathbf{T} - \lambda \mathbf{1}\right) = 0. \tag{S1.14}$$

Thus,

$$\lambda^2 - \left(t_m^{AA} + t_m^{BB}\right)\lambda + 1 = 0. \quad \text{(S1.15)}$$

The two roots of equation (S1.15) must satisfy the following relation

$$\lambda_A \lambda_B = 1, \lambda_A + \lambda_B = t_m^{AA} + t_m^{BB}. \quad \text{(S1.16)}$$

With the assumption that one root is $\lambda_A = e^{-jk_x d}$, the other root must be $\lambda_B = e^{jk_x d}$, in which k_x is the propagation factor of the Floquet mode. And finally the dispersion equation of the unbounded HCGs can be written as

$$\cos\left(\kappa_{xm} d\right) = \frac{t_m^{AA} + t_m^{BB}}{2}, \quad \text{(S1.17)}$$

in which κ_{xm} is the Bloch wave vector. Substituting equations (S1.10)-(S1.12) into equation (S1.17), we can obtain equation (1) in the main text.

It is known that for each eigenvalue λ_i, there is a corresponding eigenvector $\mathbf{U}_i$ satisfying the following equation

$$\mathbf{T}\mathbf{U}_i = \lambda_i \mathbf{U}_i. \quad \text{(S1.18)}$$

According to the transmission line network theory, the voltage and current vectors can be represented by a linear combination of the two eigenvectors. For each eigen excitation

$$\mathbf{U} = \begin{bmatrix} V_m^0 \\ I_m^0 \end{bmatrix}, \quad \text{(S1.19)}$$

$$\begin{bmatrix} V_m^B \\ I_m^B \end{bmatrix} = \lambda_i \begin{bmatrix} V_m^0 \\ I_m^0 \end{bmatrix}. \quad \text{(S1.20)}$$

Then the voltage and current column vector within the i-th ($i = A, B$) block can be expressed as

$$\begin{bmatrix} V_m^A \\ I_m^A \end{bmatrix} = \begin{bmatrix} \cos\left(k_{xm}^A x\right) & -jZ_m^A \sin\left(k_{xm}^A x\right) \\ -jY_m^A \sin\left(k_{xm}^A x\right) & \cos\left(k_{xm}^A x\right) \end{bmatrix} \begin{bmatrix} V_m^0 \\ I_m^0 \end{bmatrix}, 0 \le x \le d_A; \quad \text{(S1.21)}$$

$$\begin{bmatrix} V_m^B \\ I_m^B \end{bmatrix} = \begin{bmatrix} \cos\left(k_{xm}^B x\right) & -jZ_m^B \sin\left(k_{xm}^B x\right) \\ -jY_m^B \sin\left(k_{xm}^B x\right) & \cos\left(k_{xm}^B x\right) \end{bmatrix} \begin{bmatrix} V_m^A \\ I_m^A \end{bmatrix}, d_A \le x \le d. \tag{S1.22}$$

Thus, the voltage and current in one arbitrary unit cell can be written as

$$V_m(x) = \begin{cases} V_m^A(x), 0 \le x \le d_A \\ V_m^B(x), d_A \le x \le d \end{cases}, \tag{S1.23}$$

$$I_m(x) = \begin{cases} I_m^A(x), 0 \le x \le d_A \\ I_m^B(x), d_A \le x \le d \end{cases}. \tag{S1.24}$$

Considering that $V_m(x)$ and $I_m(x)$ are periodic functions with period d, they can be expanded in Fourier series as

$$V_m(x) = \sum_{n=-\infty}^{+\infty} V_{mn} e^{-jk_{xn}x}, 0 \le x \le d \tag{S1.25}$$

$$I_m(x) = \sum_{n=-\infty}^{+\infty} I_{mn} e^{-jk_{xn}x}, 0 \le x \le d \tag{S1.26}$$

in which V_{mn} and I_{mn} are the amplitudes of the n-th space harmonic for the m-th mode voltage $V_m(x)$ and mode current $I_m(x)$ and can be derived in the following form for the TM mode

$$I_{mn} = \frac{1}{d}\int_0^d I_m(x) e^{jk_{xn}x} dx = \frac{1}{d}\left(Q_{mn}^A + Q_{mn}^B\right), \tag{S1.27}$$

$$V_{mn} = \frac{1}{d}\int_0^d V_m(x) e^{jk_{xn}x} dx = \frac{1}{d}\left(\frac{\beta_m}{\omega\varepsilon_0\varepsilon_A} Q_{mn}^A + \frac{\beta_m}{\omega\varepsilon_0\varepsilon_B} Q_{mn}^B\right), \tag{S1.28}$$

in which

$$Q_{mn}^A = \frac{Y_m^A f_m^A \left[e^{j\left(k_{xn} - k_{xm}^A\right)d_A} - 1\right]}{j\left(k_{xn} - k_{xm}^A\right)} + \frac{Y_m^A g_m^A \left[e^{j\left(k_{xn} + k_{xm}^A\right)d_A} - 1\right]}{j\left(k_{xn} + k_{xm}^A\right)}, \tag{S1.29}$$

$$Q_{mn}^{B}=\frac{Y_{m}^{B}e^{jk_{xn}d_{A}}f_{m}^{B}\left[e^{j\left(k_{xn}-k_{xm}^{B}\right)d_{B}}-1\right]}{j\left(k_{xn}-k_{xm}^{B}\right)}+\frac{Y_{m}^{B}e^{jk_{xn}d_{A}}g_{m}^{B}\left[e^{j\left(k_{xn}+k_{xm}^{B}\right)d_{B}}-1\right]}{j\left(k_{xn}+k_{xm}^{B}\right)}, \tag{S1.30}$$

$$f_{m}^{A}=\frac{V_{m}^{0}+I_{m}^{0}/Y_{m}^{A}}{2}, \tag{S1.31}$$

$$g_{m}^{A}=\frac{V_{m}^{0}-I_{m}^{0}/Y_{m}^{A}}{2}, \tag{S1.32}$$

$$f_{m}^{B}=\frac{V_{m}^{0}+I_{m}^{0}/Y_{m}^{B}}{2}, \tag{S1.33}$$

$$g_{m}^{B}=\frac{V_{m}^{0}-I_{m}^{0}/Y_{m}^{B}}{2}. \tag{S1.34}$$

From above analysis, it can be seen that the eigensolutions of the periodic structure satisfy the Floquet condition and can be represented as the summation of infinite space harmonics. Due to the spatial periodicity of the HCGs in the x direction, all space harmonics are generally excited everywhere in the whole structure. The eigenfunctions in Region I can be simply represented by an infinite number of transmission lines, each of which stands for one space harmonic. Thus, the whole structure can be modeled by an equivalent multimode network shown in Fig. 1(b) in the main text.

The key step for the boundary value problem is the construction of a general solution for the periodic regions. Since the eigenmodes in the unbounded HCGs have been determined, the transfer relationship of admittance matrix for each region can be obtained by using the boundary condition that the tangential components of the electromagnetic fields are continuous at the interface $z=0$.

Supposing that Y_{dn}^{nm} , which is an element of the output matrix $\mathbf{Y}_{dn}$ of the HCGs layer, is known, the electromagnetic fields in Region II can be expressed as

$$\mathbf{H}_{tn}^{II}(z=0)=\sum_{m=-\infty}^{+\infty}Y_{dn}^{nm}\left[\mathbf{i}_z\times\mathbf{E}_{tn}^{II}(z=0)\right],\tag{S1.35}$$

in which $\mathbf{E}_{tn}^{II}$ and $\mathbf{H}_{tn}^{II}$ represent the n-th space harmonic electric and magnetic fields in the periodic HCGs (Region II) respectively. The directions of the electromagnetic fields for the TM modes can be denoted by $\mathbf{E}_{tn}=\mathbf{i}_x E_{tn}$ and $\mathbf{H}_{tn}=\mathbf{i}_y H_{tn}$ ($\mathbf{i}_x$, $\mathbf{i}_y$ and $\mathbf{i}_z$ are the unit vectors along the x, y and z directions respectively).

The transverse fields in the HCGs region $0\le z\le h$ for the TM modes can be expressed as modal summation with suppressing the constant vectors,

$$E_t^{II}(x,z)=\sum_{l=-\infty}^{+\infty}V_l(x)\left[a_l e^{-j\beta_{ml}(z+h)}+b_l e^{j\beta_{ml}(z+h)}\right],\tag{S1.36}$$

$$H_t^{II}(x,z)=\sum_{l=-\infty}^{+\infty}I_l(x)\left[a_l e^{-j\beta_{ml}(z+h)}-b_l e^{j\beta_{ml}(z+h)}\right],\tag{S1.37}$$

the n-th Fourier component is

$$E_{tn}^{II}(x,z)=\sum_{l=-\infty}^{+\infty}V_{nl}(x)\left[a_l e^{-j\beta_{ml}(z+h)}+b_l e^{j\beta_{ml}(z+h)}\right],\tag{S1.38}$$

$$H_{tn}^{II}(x,z)=\sum_{l=-\infty}^{+\infty}I_{nl}(x)\left[a_l e^{-j\beta_{ml}(z+h)}-b_l e^{j\beta_{ml}(z+h)}\right],\tag{S1.39}$$

in which a_l and b_l are the forward and backward traveling wave amplitudes of l-th Floquet mode in the HCGs respectively and β_{ml} is the wave vector along the z direction. V_{nl} and I_{nl} are the amplitudes of the n-th space harmonic for $V(x)$ and $I(x)$ respectively.

Since the interface at $z=h$ is a PEC plane, the coefficient column vectors $\mathbf{a}=\left[a_{-\infty},\cdots,a_0,\cdots,a_{+\infty}\right]^{\mathrm{T}}$ and $\mathbf{b}=\left[b_{-\infty},\cdots,b_0,\cdots,b_{+\infty}\right]^{\mathrm{T}}$ satisfying

$$\mathbf{b}=\mathbf{\Gamma}\mathbf{a},\tag{S1.40}$$

where $\mathbf{\Gamma}$=-**1** (**1** is the unit matrix) is the reflection coefficient matrix due to the PEC plane.

Thus, according to equations (S1.35), (S1.38)-(S1.40), the input admittance matrix $\mathbf{Y}_{dn}$ looking down into Region II at the interface $z=0$ can be derived as

$$\mathbf{Y}_{dn}=\mathbf{I}\left(\mathbf{1}-e^{i\beta_m h}\mathbf{\Gamma}e^{i\beta_m h}\right)\left(\mathbf{1}+e^{i\beta_m h}\mathbf{\Gamma}e^{i\beta_m h}\right)^{-1}\mathbf{V}^{-1}, \tag{S1.41}$$

meanwhile, it is easy to determine the input admittance matrix $\mathbf{Y}_{up}$ looking up into Region I at the interface $z=0$, which is a diagonal matrix as

$$\mathbf{Y}_{up}=\left(\delta_{nl}Y_n\right), \tag{S1.42}$$

in which $Y_n=\omega\varepsilon_0/k_{zn}$ for the TM mode, $k_{zn}=\sqrt{k_0^2-k_{xn}^2}$ and $k_{xn}=k_x+2n\pi/d\left(n=0,\pm1,\pm2,\cdots\right)$.

Therefore, the complex eigenvalue of the proposed structure can be finally solved by the generalized transverse resonance condition at $z=0$ interface

$$\det\left(\mathbf{Y}_{up}+\mathbf{Y}_{dn}\right)=0. \tag{S1.43}$$

Thus, we can finally obtain equation (5) in the main text by solving equation (S1.43) based on the assumptions that $\lambda_0\gg d>d_A,d_B$ and $k_x>k_0$. Only the fundamental surface mode (m=0) is kept with all high-order diffraction effects neglected.

2. Derivation of the dispersion relation of 2D HCGs-based SSPs with effective medium approximation

In this section, we will show how the same dispersion relation can be obtained by using the effective medium approximations. Supposing a plane wave is incident on the surface of the three layered structure with air on the top, PEC at the bottom, and a homogeneous but anisotropic medium layer of h in between with the electromagnetic parameters given by $\left[\bar{\bar{\varepsilon}}\right]$ and $\left[\bar{\bar{\mu}}\right]$ (denoted in equations (6) and (7) in the main text) shown in Fig. 1(c) in the main text and the

only considered mode is the fundamental surface mode (m=0), which is characterized by the perpendicular wave vector β_0. The reflection and transmission coefficients for the TM mode from layer i' to j' are given by[1]

$$r_{i'j'} = \frac{k_{z,j'}/\eta_{i'} - k_{z,i'}/\eta_{j'}}{k_{z,j'}/\eta_{i'} + k_{z,i'}/\eta_{j'}}, \tag{S2.1}$$

$$t_{i'j'} = \frac{2k_{z,j'}/\eta_{i'}}{k_{z,j'}/\eta_{i'} + k_{z,i'}/\eta_{j'}}, \tag{S2.2}$$

in which $\eta_{i'} = \sqrt{\mu_{y,i'}/\varepsilon_{x,i'}}$ is the wave impedance, $\varepsilon_{x,i'}$ is the x-component of the permittivity, and $\mu_{y,i'}$ is the y-component of the effective permeability in medium i', respectively. $k_{z,j'}$ is the z-component of the wave vector in medium j' and can be written as $k_{z,j'} = \sqrt{k_0^2\varepsilon_{x,j'}\mu_{y,j'} - k_x^2}$. The value of $r_{i'j'}$ is always -1 when medium j' is a PEC, indicating complete reflection of energy with a π phase shift of the electric field.

To obtain the reflection coefficient R, we apply the transfer matrix formalism[2], in which the 2×2 matrix $D_{i'j'}$ describes the $i'-j'$ interface and $\Phi_{l'}$ describes the propagation through medium l'.

$$D_{i'j'} = \frac{1}{t_{i'j'}}\begin{pmatrix} 1 & r_{i'j'} \\ r_{i'j'} & 1 \end{pmatrix}, \tag{S2.3}$$

$$\Phi_{l'} = \begin{pmatrix} e^{ik_{z,l'}h} & 0 \\ 0 & e^{-ik_{z,l'}h} \end{pmatrix}. \tag{S2.4}$$

The total transfer matrix to describe the three layer structures in Fig. 1(c) in the main text can then be written as $M = D_{12}\Phi_2 D_{23}$, where the subscripts 1, 2, and 3 represent the air, the

homogeneous layer and the continuous PEC layer, respectively. The specular reflection coefficient R can be obtained from M by $R = M_{2,1}/M_{1,1}$, where $M_{i'',j''}$ is the component of M identified by row i'' and column j''

$$R = \frac{(\varepsilon_x k_z - \beta_0) + (\beta_0 + \varepsilon_x k_z) e^{i2\beta_0 h}}{(\varepsilon_x k_z + \beta_0) - (\beta_0 - \varepsilon_x k_z) e^{i2\beta_0 h}}, \tag{S2.5}$$

in which k_z and β_0 are the wave vector component in the air and homogeneous medium respectively (*i.e.* $k_z = k_{z,1}$, $k_{z,2} = \beta_0$ and $k_{z,3} = 0$).

Thus, when $k_x > k_0$ ($k_z = i\sqrt{k_x^2 - k_0^2}$), we can finally obtain equation (9) in the main text by setting the denominator of equation (S2.5) to zero.

Supplementary References

1. Inan, U. S. and Inan, A. S. Electromagnetic Waves (Prentice Hall, 2000).
2. Yeh, P. Optical Waves in Layered Media (Wiley, 2005).
